# Correlation between PFGE Groups and *mrp/epf/sly* Genotypes of Human *Streptococcus suis* Serotype 2 in Northern Thailand

**DOI:** 10.1155/2014/350416

**Published:** 2014-03-06

**Authors:** Prasit Tharavichitkul, Kanreuthai Wongsawan, Naoki Takenami, Sumalee Pruksakorn, Achara Fongcom, Marcelo Gottschalk, Banyong Khanthawa, Volaluk Supajatura, Shinji Takai

**Affiliations:** ^1^Department of Microbiology, Faculty of Medicine, Chiang Mai University, Chiang Mai 50200, Thailand; ^2^Department of Veterinary Biosciences and Public Health, Faculty of Veterinary Medicine, Chiang Mai University, Chiang Mai 50200, Thailand; ^3^Department of Animal Hygiene, School of Veterinary Medicine and Animal Sciences, Kitasato University, Towada, Aomori 034-8628, Japan; ^4^Department of Medicine, Lamphun Provincial Hospital, Lamphun 51000, Thailand; ^5^Faculty of Veterinary Medicine, University of Montreal, Saint-Hyacinthe, QC, Canada J2S 2M2; ^6^Microbiology Laboratory, Maharaj Nakorn Chiang Mai Hospital, Chiang Mai 50200, Thailand

## Abstract

*Streptococcus suis* infection is a severe zoonotic disease commonly found in Northern Thailand where people often consume raw pork and/or pig's blood. The most frequent clinical presentations are meningitis, sepsis, and endocarditis with higher rate of mortality and hearing loss sequelae. To clarify the correlation between pulsed-field gel electrophoresis (PFGE) groups and *mrp/epf/sly* genotypes of *S. suis* serotype 2, 62 patient and 4 healthy pig isolates from Northern Thailand were studied. By PFGE analysis, at 66% homology, most human isolates (69.4%) and 1 pig isolate were in group A, whereas 14.5% of human isolates and 3 out of 4 pig isolates were in group D. According to *mrp/epf/sly* genotypes, 80.6% of human isolates were identified in *mrp* ^+^ *epf* ^−^ *sly* ^−^ and only 12.9% were in *mrp* ^−^ *epf* ^−^ *sly* ^+^ genotypes; in contrast, 1 and 3 pig isolates were detected in these two genotypes, respectively. Interestingly, all isolates of *S. suis* serotype 2 classified in PFGE groups A, B, and E were set in *mrp* ^+^ *epf* ^−^ *sly* ^−^ genotypes. These data show a close correlation between PFGE groups and *mrp/epf/sly* genotypes of human *S. suis* serotype 2.

## 1. Introduction

*Streptococcus suis*, recognized as a significant swine and human pathogen, mainly causes meningitis, sepsis, endocarditis, and septic shock [1]. It can be transmitted to humans by contact with sick or carrier pigs, pig-derived products [2], or eating undercooked pork [3, 4]. Capsular polysaccharide (CPS) is the most important proven critical virulence factor, due to its antiphagocytosis activity [5]. Of 35 serotypes, serotype 2 is the most frequently isolated and associated with disease in both animals and humans [6]. In addition, virulence-related proteins, such as muramidase-released protein (MRP), extracellular factor (EF), and hemolysin (suilysin, SLY), are expressed by some strains of* S. sui* “as discussed by Gottschalk and Segura [1].” These proteins are encoded by the genes* mrp*,* epf*, and* sly*, respectively. MRP/EF/SLY phenotypes or* mrp/epf/sly* genotypes of* S. suis *serotype 2 have been studied mostly in pig isolates with very little data for human isolates [1, 7], especially in Northern Thailand. Pulsed-field gel electrophoresis (PFGE) of DNA restricted with SmaI has been confirmed to be valuable for evaluating the genetic diversity of* S. suis* [8]. The present study aims to clarify the correlation between PFGE and* mrp/epf/sly* genotypes of* S. suis* serotype 2 isolated from patients in Northern Thailand.

## 2. Materials and Methods

### 2.1. *S. suis* Serotype 2 Strains from Humans and Healthy Pigs

A total of 66* S. suis* serotype 2 (SS2) isolates were included in this study. Sixty-two human SS2 were isolated from 43 and 19 patients at Maharaj Nakorn Chiang Mai Hospital and Lamphun Provincial Hospital, respectively. To isolate* S. suis* from pigs, a total of 150 submaxillary lymph nodes of healthy pigs that were bought from two retail markets in the municipal area of Chiang Mai province between November and December 2002 were studied. From each homogenized sample,* S. suis* was isolated on NNCC agar (sodium azide, nalidixic acid, colistin-crystal violet agar) “as described by Kataoka et al. [9]” and selective blood agar (sheep blood agar + SR126E, oxoid). All *α*-hemolytic gram-positive cocci were screened first with standard biochemical tests and confirmed with an API 20 Strep (bioMérieux).* S. suis* serotype 2 was identified by the coagglutination test with antiserotype 1 (negative reaction) and 2 (positive reaction) antibodies “as discussed by Gottschalk et al. [6].” P1/7 strain, a reference strain of serotype 2, which was isolated from a diseased pig with the* mrp* ^*+*^ *epf* ^*+*^ *sly* ^*+*^ genotype, was used as a positive control. For negative control, we used beta-hemolytic streptococci and distilled water.

### 2.2. Characterization of the Genetic Diversity of* S. suis* Isolates by PFGE

This method was modified from Son et al. [10]. For each* S. suis* serotype 2 strain, two independent extractions of DNA were performed to verify the reproducibility of patterns. Briefly, the cell suspension was prepared from overnight Todd-Hewitt Broth (Difco) culture and mixed with 1% agarose to form the agarose plugs. The cells were lysed with lysozyme and then treated with proteinase. The DNA was digested with SmaI and its fragments were resolved by PFGE within 1% electrophoresis grade agarose gel using a CHEF-DR II system (Bio-Rad). The gels were stained with ethidium bromide and photographed under a UV light. The cluster analysis was provided by Bionumeric software (Applied Maths) and transformed into an agglomerative cluster using the unweighted pair group method with arithmetic averages (UPGA) (based on Dice coefficients).

### 2.3. Detection of* mrp*,* epf*, and* sly* Putative Virulent Genes by PCR

Chromosomal DNA isolations were performed “as described by Pruksakorn et al. [11].” Specific oligonucleotide primers for the* mrp* gene were designed as FWM (5′-GAGAGGAACTGATACGA-3′) and REM (5′-CCAAGAGCTGACTTAGGA-3′). The amplified fragment was 515 base pairs. Specific oligonucleotide primers were used for the epf, epf,* and* sly *genes with amplified fragments 626 bp, 1,278–2,993 bp, [12], and 1,282 bp, [13] respectively.

Amplification reaction for each gene was individually performed in a Perkin Elmer Cetus DNA thermal cycler with the reaction mixtures including the four deoxynucleotide triphosphates, each primer, and* Taq* DNA polymerase. The PCR products were run in a 2% agarose gel (*mrp*,* epf*, and epf*) or a 0.8% agarose gel (*sly*), stained with ethidium bromide, and visualized by UV transillumination. Lambda DNA digested with* EcoRI* and* HindIII* and 100 bp DNA ladder markers (all from Promega Co.) were used to estimate fragment size.

## 3. Results

### 3.1. Isolation of* S. suis* from Submaxillary Lymph Nodes of Healthy Pigs

Of 150 submaxillary lymph node samples, 68* S. suis* isolates (45.3%) were recovered. However, only 6 isolates (4%), detected from 4 samples (2.6%; 3 isolates were recovered from the same sample), were confirmed to belong to serotype 2. Hence, the four isolates recovered from 4 animals were included in this study.

### 3.2. Characterization of the Genetic Diversity of* S. suis* Isolates by PFGE

**Figure 1:**
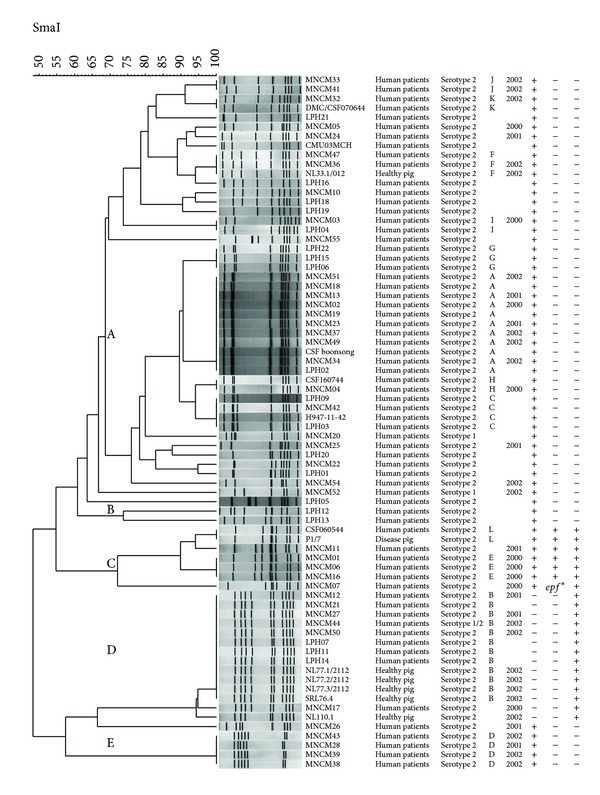
Dendrogram showing the relationship between PFGE groups,* mrp-epf-sly* genotypes of* S. suis* serotype 2 isolates, and P1/7 reference strain.

Reproducible results were observed because an identical pattern was shown for each* S. suis* isolate after the two independent DNA extractions (results not shown). PFGE patterns after digestion with SmaI were characterized by 6 to 10 major bands with sizes ranging from 48.5 to 436 kb. Of the 66 isolates, cluster analysis by Bionumerics software revealed 5 PFGE groups (A to E), at 66% similarity (Figure 1 and Table 1). Most patient isolates (43 of 62 or 69.4%) were expressed in PFGE group A, followed by PFGE group D (9 of 62 or 14.5%). The 4 pig isolates presented 2 different PFGE groups (1 in A and 3 in D) and the reference strain P1/7 belonged to pulsotype l group C.

### 3.3. Detection of* mrp*,* epf*, and* sly* Putative Virulent Genes by PCR

When the three genes were composed as* mrp/epf*/*sly* genotype, 3 different types were analyzed (Table 1). Most of the* S. suis* serotype 2 isolates from humans (50 of 62 or 80.6%) and only 1 pig isolate presented the* mrp* ^*+*^ *epf* ^−^ *sly* ^−^ genotype whereas 3 of the 4 pig isolates presented the* mrp* ^−^ *epf* ^−^ *sly* ^*+*^ genotype, which was also found in 8 human isolates. Four human isolates with the* mrp* ^*+*^ *epf* ^*+*^ *sly* ^*+*^ genotype corresponded to the genotype of a P1/7 reference strain.

### 3.4. Correlation between PFGE Groups and* mrp/epf/sly* Genotypes

The association between PFGE groups and* mrp/epf/sly* genotypes shows that all isolates of SS2 in PFGE group A, B, and E were classified in the* mrp* ^+^ *epf* ^−^ *sly* ^−^ genotype. Most of the PFGE group D strains belong to the* mrp* ^−^ *epf* ^−^ *sly* ^*+*^ genotype, except for 1. All isolates of PFGE group C belong to* mrp* ^+^ *epf* ^+^ *sly* ^+^ or* mrp* ^+^ epf* sly ^+^ genotypes (Table 1).

## 4. Discussion

PFGE is a high-resolution genotypic technique originally proved by Allgaier et al. [14] to be most suitable for differentiating single isolates, thus making it the most reliable in epidemiological studies with* S. suis*. Berthelot-Hérault et al. [8] confirmed the PFGE analysis to be a very useful tool for such investigations. They reported on the SmaI restriction PFGE patterns (A, B, and C) of 123* S. suis* isolates from pigs in France and from humans worldwide. In the present study, while most of* S. suis* serotype 2 isolates from humans were identified as PFGE groups A and D (69.4% and 14.5%, resp.), isolates from healthy pigs were included in group A and D (1 and 3 isolates, resp.). Interestingly,* S. suis* isolates from humans and healthy pigs were clustered in the same PFGE subgroups (“b” and “f”), except for 1 pig isolate (NL 110.1). This evidence may raise the possibility that healthy carrier pigs could transmit* S. suis* to humans.

The MRP/EF/SLY phenotypes and/or* mrp/epf/sly *genotypes of* S. suis* serotype 2 have been used as a tool to determine genetic diversity among human and pig isolates as well as in epidemiological studies. In addition, a clear association between either MRP^+^, EF^+^, SLY^+^ or* mrp* ^+^,* epf* ^+^,* sly* ^+^ phenotypes/genotypes with virulence has largely been established in many countries. Most* S. suis* invasive serotypes 2 strains isolated from diseased pigs in Europe were included in the* mrp* ^*+*^ */mrp* ^*s*^ *epf* ^*+*^ /epf*/sly ^*+*^ genotype [15]. Most serotype 2 strains isolated from ill pigs or human patients in China and Vietnam also present such a* mrp* ^+^,* epf* ^+^,* sly* ^+^ phenotype [16, 17]. However, Gottschalk et al. [6] first described a high frequency of MRP^−^EF^−^SLY^−^ phenotype of most swine strains (72%) and two human isolates. In addition, 16 strains (18.8%) from diseased pigs were MRP^+^/MRP* EF^−^SLY^−^. These phenotypes seem to be common in North America, since similar results were confirmed with strains recovered from diseased pigs in the United States [18].

However, the situation seems to be different in Thailand. Our results are similar to those presented by Takamatsu et al. [7] who studied the* mrp/epf*/*sly* genotypes of 19 human SS2 isolates from Thailand, showing that most of them (68.4%) were assigned to* sly* ^−^ *epf* ^−^ *mrp* ^∗∗+^ genotypes. Healthy pig isolates from this study showed the same genotypes as the human isolates (either* mrp* ^*+*^ *epf* ^−^ *sly* ^−^ or* mrp* ^−^ *epf* ^−^ *sly* ^*+*^ genotypes). Moreover, the 3 isolates from the same pig's lymph node all showed the same PFGE pulsotype b and* mrp* ^−^ *epf* ^−^ *sly* ^*+*^ genotype. This usually indicates that this animal is probably still recovering from its illness. It is interesting to note that the reference isolate P1/7 showed a high genetic similarity with a human isolate, CSF060544 (PFGE group C pulsotype l and* mrp* ^+^,* epf* ^+^,* sly* ^+^ phenotype). This may indicate the possible transmission of diseased pig isolate to human.

Our team, Wongsawan et al. [19] had analyzed the genetic diversity of* S. suis* isolated from humans by both PFGE and* mrp/epf/sly* genotypes; however, they have not been compared one each other. In the present study, we wanted to address the correlation between different groups and genotypes of* S. suis *serotype 2 for future epidemiological studies. Interestingly, we found that all isolates of* S. suis* serotype 2 classified in PFGE groups A, B, and E were included in the* mrp* ^*+*^ *epf* ^−^ *sly* ^−^ genotype. In addition, all isolates in PFGE groups C and D except one were expressed in the genotypes* mrp* ^*+*^ *epf* ^*+*^ /epf* sly ^*+*^ and* mrp* ^−^ *epf* ^−^ *sly* ^*+*^, respectively. Kerdsin et al. [20] reported the significant association between ST1 strain and the meningitis category (*P* < 0.0001); they did not discuss the correlation between PFGE groups and clinical features. In our disease outcomes (Table 2), we found that most of Bacteremia (24 of 30 or 80.0%) and meningitis (13 of 19 or 68.4%) cases were in PFGE group A. Fascinatingly, all 3 cases of toxic shock syndrome were also included in PFGE group A. For endocarditis, each 3 of 10 (30%) cases were distributed in PFGE groups A, D, and E.

In conclusion,* mrp/epf/sly* genotypes of* S. suis* serotype 2 isolated from humans are strongly correlated with PFGE analysis. The few isolates from pigs analyzed in this study were similar to those isolated from patients. It will be necessary to further study a higher number of swine isolates recovered from ill animals in Thailand.

## Figures and Tables

**Table 1:** Correlation between PFGE groups and *mrp*-*epf*-*sly* genotypes of *S. suis* serotype 2 isolated from 62 patients and 4 healthy pigs.

PFGE groups (number found)	*mrp-epf-sly* genotypes (number found)	Patient isolates (% found)	Healthy pig isolates (% found)
A (44)	*mrp* ^ +^ *epf* ^−^ *sly* ^−^ (44)	43 (69.4)	1 (25)
B (2)	*mrp* ^ +^ *epf* ^−^ *sly* ^−^ (2)	2 (3.2)	0
C (4)	*mrp* ^ +^ *epf* ^ +^/epf*sly ^+^ (4)	4 (6.4)	0
D (12)	*mrp* ^−^ *epf* ^−^ *sly* ^ +^ (11)	8 (12.9)	3 (75)
	*mrp* ^ +^ *epf* ^−^ *sly* ^−^ (1)	1 (1.6)	0
E (4)	*mrp* ^ +^ *epf* ^−^ *sly* ^−^ (4)	4 (6.4)	0

*ep* f*: When used the same primer with the *epf* gene for PCR detection, the amplified fragment of this gene was approximately 1,278–3,000 bp.

**Table 2:** Correlation between PFGE groups and *S. suis* serotype 2 infections.

PFGE groups	Bacteremia	Meningitis	Endocarditis	Toxic shock syndrome	Total
A	24	13	3	3	43
B	2	0	0	0	2
C	0	3	1	0	4
D	4	2	3	0	9
E	0	1	3	0	4
Total	**30**	**19**	**10**	**3**	**62**
